# Effect of cessation of GH treatment on cognition during transition phase in Prader-Willi syndrome: results of a 2-year crossover GH trial

**DOI:** 10.1186/s13023-016-0535-7

**Published:** 2016-11-16

**Authors:** R. J. Kuppens, E. F. Mahabier, N. E. Bakker, E. P. C. Siemensma, S. H. Donze, A. C. S. Hokken-Koelega

**Affiliations:** 1 Dutch Growth Research Foundation, Rotterdam, The Netherlands; 2 Department of Pediatrics, Erasmus University Medical Center-Sophia Children’s Hospital, Subdivision of Endocrinology, Westzeedijk 106, 3016 AH Rotterdam, The Netherlands

**Keywords:** Prader-Willi syndrome, Growth hormone treatment, Cognition, Young adults

## Abstract

**Background:**

Patients with Prader-Willi syndrome (PWS) have a cognitive impairment. Growth hormone (GH) treatment during childhood improves cognitive functioning, while cognition deteriorates in GH-untreated children with PWS. Cessation of GH treatment at attainment of adult height (AH) might deteriorate their GH-induced improved cognition, while continuation might benefit them. We, therefore, investigated the effects of placebo versus GH administration on cognition in young adults with PWS who were GH-treated for many years during childhood and had attained AH.

**Method:**

Two-year, randomized, double-blind, placebo-controlled cross-over study in 25 young adults with PWS. Cross-over intervention with placebo and GH (0.67 mg/m^2^/day), both during 1 year.

**Results:**

Total (TIQ), verbal (VIQ) and performance IQ (PIQ) did not deteriorate during 1 year of placebo, compared to GH treatment (*p* > 0.322). Young adults with a lower TIQ had significantly more loss of TIQ points during placebo versus GH, in particular VIQ decreased more in those with a lower VIQ. The effect of placebo versus GH on TIQ, VIQ and PIQ was not different for gender or genotype.

**Conclusions:**

Compared to GH treatment, 1 year of placebo did not deteriorate cognitive functioning of GH-treated young adults with PWS who have attained AH. However, patients with a lower cognitive functioning had more loss in IQ points during placebo versus GH treatment. The reassuring finding that 1 year of placebo does not deteriorate cognitive functioning does, however, not exclude a gradual deterioration of cognitive functioning on the long term.

**Trial registration:**

ISRCTN24648386, NTR1038, Dutch Trial Register, www.trialregister.nl. Registered 16 August 2007.

## Background

Prader-Willi Syndrome (PWS) is a neurogenetic disorder resulting from the lack of expression of the PWS region on the paternally derived chromosome 15, caused by paternal deletion, maternal uniparental disomy (mUPD), imprinting center defect (ICD) or balanced translocation [1]. PWS is characterized by a number of symptoms, such as muscular hypotonia, short stature, abnormal body composition with high fat mass and low lean body mass, severe hyperphagia, behavioral problems and cognitive impairment [1–3].

MRI studies in PWS suggested that lower cortical complexity partially underlies cognitive impairment and developmental delay [4], with structural brain abnormalities and different neurodevelopmental patterns between children with a deletion and an mUPD [5]. Certain cognitive skills improved significantly during GH treatment, while GH-untreated children with PWS showed a deterioration of cognitive functioning [6, 7]. Children with an mUPD started off with lower visuospatial skills, but showed a significant improvement during 4 years of growth hormone (GH), resulting into a similar cognitive functioning in all genotypes [6].

GH treatment has also positive effects on body composition, bone mineral density, psychomotor development, adaptive functioning, linear growth and adult height (AH) [6–11]. As a result, GH treatment has substantially changed the phenotype of children with PWS [8, 11], but when they attain AH, they have to stop GH treatment because most do not fulfill the criteria of adult GH deficiency. There are no studies about cognition after stop of GH treatment in this new generation of PWS patients. In untreated adults with PWS, lower IGF-I levels were correlated with poorer intellectual skills [12], which might suggest that discontinuation of GH treatment, which decreases IGF-I, could be disadvantageous.

Given the positive effects of GH and IGF-I levels on cognition in children with PWS, we hypothesized that the cognition in young adults with PWS would deteriorate after cessation of GH treatment compared to the continuation of GH administration. We, therefore, investigated the effects of placebo versus GH on cognition in young adults with PWS who had attained AH, in a 2-year, randomized, double-blind, placebo-controlled cross-over study.

## Methods

### Subjects

Inclusion criteria of the present study were (1) genetically confirmed diagnosis of PWS; (2) GH treatment during childhood for at least 2 years and being on GH at time of inclusion; and (3) AH attainment, defined as a height velocity less than 0.5 cm per 6 months and complete epiphyseal fusion. Exclusion criteria were (1) use of medication to reduce weight; (2) non-cooperative behavior; or (3) inability to perform cognitive tests. Due to the last exclusion criterion, 2 patients could not participate; 1 due to poor cognitive skills and a severe hearing impairment, and with an IQ of 58 who refused to speak in the hospital. From June 2008 to January 2014, 33 young adults fulfilled the inclusion criteria. Two did not want to continue GH-injections and 3 parents refused participation due to too large burden of hospital visits. Twenty-eight young adults (8 boys, 20 girls) with PWS aged 14.1–20.2 years were included in the GH/placebo study. One participant died due to gastric rupture 3 months after start while receiving placebo. Twenty-five young adults completed the present study.

During childhood, the standard GH dose was 1 mg/m^2^/day. In the present study during transition from childhood into adulthood, GH dose was set lower at a fixed dose of 0.67 mg/m^2^/day (≈0.023 mg/kg/day). We did neither titrate on serum IGF-I levels nor on body composition. Twelve (48%) young adults used sex steroid replacement therapy, 7 (28%) thyroid hormone supplementation, 2 (8%) modafinil and 1 (4%) risperidone and citalopram. All patients were on a strict diet and an exercise program.

### Design

Two-year, randomized, double-blind, placebo-controlled, cross-over study investigating the effects of 1 year placebo versus 1 year GH on cognitive functioning. The duration per phase was 1 year in order to prevent retesting phenomenon. A clinically relevant deterioration of cognitive functioning was arbitrarily defined as a decrease of 5 IQ points, taking into account the significant improvement of IQ during 4 years GH treatment in children with PWS [6]. Young adults were stratified according to gender and BMI (below/above 25 kg/m^2^) and then randomly and blindly assigned to receive 1 year of subcutaneous injections once daily at bedtime of either 0.67 mg/m^2^/day GH (Genotropin®, 5 mg/ml, Pfizer) or 1 year of identical appearing placebo (placebo, Pfizer), after which they crossed-over to the alternative treatment for another year. An independent statistician generated the random allocation sequence. Investigators were blinded for the allocation. An independent physician monitored the safety during the study. During the entire study period, unblinding was not necessary.

### Measurements

Patients were 3-monthly seen by the PWS-team of the Dutch Growth Research Foundation in collaboration with pediatric endocrinologists and pediatricians. At each visit, the injection dose was adjusted to the calculated body surface area. In addition, patients visited the Sophia’s Children Hospital every 6 months and at baseline, 12 and 24 months the following data were obtained: cognitive functioning, anthropometric measurements, fasting blood levels of IGF-I, and (severe) adverse events.

All cognitive measurements were performed by a psychologist experienced in testing young adults with PWS. The 11 recommended subscales of Wechsler Adult Intelligence Scale 3^rd^ Edition (WAIS-III) were used to assess total IQ (TIQ) in patients over 16 years of age [13]. Verbal IQ (VIQ) subtests were Vocabulary, Similarities, Arithmetic, Digit Span, Information and Comprehension. Performance IQ (PIQ) subtests were Picture Completion, Coding, Block design, Matrix Reasoning and Picture Arrangement. The 10 recommended subscales of Wechsler Intelligence Scale for Children 3^rd^ Edition (WISC-III) were used to assess TIQ in 4 patients younger than 16 years [14]. VIQ subtests were Information, Similarities, Arithmetic, Vocabulary and Comprehension. PIQ subtests were Picture Completion, Coding, Picture Arrangement, Block Design and Visual Puzzles. It was reported that WISC IQ and WAIS IQ are comparable in 16 year old young adults [15]. In both tests, scores on all subtests were expressed as standard deviation scores, based on Dutch population data for the same age [13, 14]. Standard subtest scores ranged from 1 (−3 SDS) to 19 (+3 SDS), with a mean of 10 (0 SDS).

Standing height was measured with a calibrated Harpenden stadiometer, weight was determined on a calibrated scale (ServoBalance KA-20-150S) and BMI was calculated. Height, weight and BMI were expressed as SDS, adjusted for age and sex [16, 17]. SDS values were calculated with GrowthAnalyser 4.0.

### Assays

Blood samples were collected after an overnight fast and measured in one laboratory. IGF-I was measured using an immunometric technique on Immulite 1000 (LKGF1, Siemens Medical Solutions Diagnostics) with an interassay variation <7.5%. Serum levels of IGF-I were expressed as SDS, adjusting for age and gender [18, 19].

### Statistics

Statistical analysis was performed with SPSS version 23.0. Calculation of sample size indicated that 22 subjects were required for a power of >80% with a significance level of 0.05. As data were not normally distributed, nonparametric tests were used and data expressed as median (interquartile range (IQR)), unless otherwise described. Statistical analysis appropriate for cross-over trials were used, taking into account any carry-over or treatment-period effect, calculated by Wilcoxon Signed Rank test and Mann Whitney U tests, but these were not found. Correlations between effect of GH treatment and other parameters were assessed using Spearman’s rho. Differences were considered significant if *p*-value was <0.05.

### Study approval

Written informed consent was obtained from patients and parents. The study protocol was approved by the Medical Ethics Committee of Erasmus University Medical Center, Rotterdam, and registered at Dutch Trial Register (www.trialregister.nl NTR1038).

## Results

### Baseline characteristics

Median age of the 25 young adults with PWS (7 boys, 18 girls) with cognitive functioning tests was 17.8 (15.7 to 18.5) years and BMI was +1.1 (−0.8 to +1.7) SDS (Table 1). Nine (36%) patients had a deletion, 13 (52%) an mUPD, 2 (8%) an ICD and 1 (4%) a translocation. At baseline (adult height (AH)), both treatment arms had similar characteristics. During childhood, GH treatment was started at a median (IQR) age of 8.8 (6.3 to 10.1) years and patients were treated for 8.6 (7.0 to 10.5) years until AH.

**Table 1:** Baseline characteristics of total group and per treatment schedule

	PWS (*n* = 25)		Placebo/GH (*n* = 12)		GH / Placebo (*n* = 13)		p*
Boys/girls (*n*)		7/18		3/9		4/9	
Genetic subtype							
- Deletion		9		2		7	
- mUPD		13		8		5	
- ICD/translocation		3		9		1	
Age (yrs)	17.8	(15.7 to 18.5)	17.2	(14.9 to 19.4)	17.8	(16.9 to 18.0)	0.852
Adult height (SDS)	−1.7	(−2.2 to–1.0)	−1.7	(−2.4 to–1.1)	−1.8	(−2.0 to −0.9)	0.852
BMI for age (SDS)	1.1	(−0.8 to 1.7)	1.3	(−0.4 to 1.7)	1.1	(−0.8 to 2.0)	0.936
BMI for age PWS (SDS)	−1.1	(−2.2 to −0.6)	−1.2	(−2.2 to −0.6)	−1.1	(−2.3 to −0.5)	0.689
Age at start GH treatment (yrs)	8.8	(6.3 to 10.1)	8.1	(5.8 to 9.8)	8.8	(6.9 to 11.2)	0.376
Duration of GH treatment (yrs)	8.6	(7.0 to 10.5)	8.7	(7.2 to 11.4)	8.3	(6.5 to 10.5)	0.650
IGF-I (SDS)	2.1	(1.7 to 3.0)	1.7	(1.1 to 3.0)	2.2	(2.0 to 3.0)	0.051
FM%	38.6	(32.3 to 44.9)	39.0	(32.4 to 45.6)	37.3	(30.5 to 44.9)	0.810
Lean body mass (kg)	36.5	(30.6 to 41.5)	35.0	(29.6 to 43.9)	37.0	(33.2 to 40.2)	0.611
Total IQ	62	(56 to 73)	60	(54 to 63)	67	(57 to 78)	0.095
Verbal IQ	65	(57 to 72)	66	(60 to 72)	65	(55 to 74)	0.713
- Vocabulary	−2.3	(−2.8 to −1.7)	−2.3	(−2.8 to −1.8)	−2.0	(−2.8 to −1.4)	0.464
- Similarities	−2.0	(−2.5 to −1.0)	−2.0	(−2.7 to −1.5)	−1.7	(−2.5 to −0.8)	0.382
- Arithmetic	−2.0	(−2.0 to −1.7)	−2.0	(−2.3 to −1.6)	−2.0	(−2.0 to −1.6)	0.635
- Digit Span	−2.0	(−2.3 to −2.0)	−2.0	(−2.0 to −1.2)	−2.3	(−2.4 to −1.9)	0.129
- Information	−1.7	(−2.0 to −1.3)	−1.7	(−1.8 to −1.3)	−1.8	(−2.0 to −1.3)	0.792
- Comprehension	−2.0	(−2.6 to −1.5)	−2.2	(−2.7 to −1.6)	−2.0	(−2.4 to −1.2)	0.792
Performance IQ	60	(54 to 72)	59	(54 to 67)	66	(52 to 74)	0.635
- Picture Completion	−2.5	(−3.0 to −1.5)	−2.5	(−2.8 to −1.7)	−2.5	(−3.0 to −1.3)	0.792
- Coding	−3.0	(−3.0 to −2.3)	−3.0	(−3.0 to −2.6)	−2.9	(−3.0 to −1.6)	0.428
- Block design	−2.2	(−2.2 to −1.3)	−2.0	(−3.0 to −1.5)	−1.5	(−1.9 to −0.8)	0.034
- Matrix Reasoning	−2.3	(−2.3 to −1.3)	−2.0	(−2.3 to −1.7)	−2.0	(−2.3 to −1.0)	0.786
- Picture Arrangement	−2.7	(−2.7 to −1.5)	−2.3	(−2.8 to −1.8)	−1.7	(−2.0 to −1.4)	0.082

Data expressed as median (IQR). *Comparison between two treatment schedules

### Cognitive Functioning at AH

Median total IQ (TIQ) at AH was 62 (56 to 73) points, with a non-significantly higher verbal IQ (VIQ) of 65 (57 to 72) points than performance IQ (PIQ) of 60 (54 to 72) points (*p* = 0.157) (Table 1).

### Placebo

**Fig. 1:**
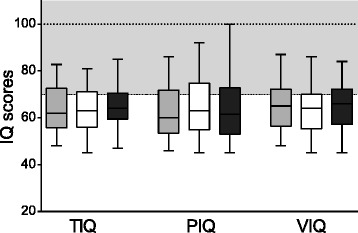
Cognitive functioning at baseline, after 1 year of GH treatment and after 1 year of placebo. Total, performance and verbal IQ at baseline (*in light grey*), after 1 year of placebo (*in white*) and after 1 year of GH treatment (*in dark grey*). Boxes represent 1^st^ and 3^rd^ quartile, with median in the middle. Whiskers indicate range. There are no significant differences

TIQ, VIQ and PIQ had not changed after 1 year of placebo (*p* = 0.422, *p* = 0.220 and *p* = 0.488) (Fig. 1). The young adults had similar scores on the 11 subtests before and after 1 year of placebo (Table 2).

**Table 2:** Cognitive function, body composition and IGF-I SDS of PWS adolescents at different stages in the study

	Treatment schedule								*p*-value
	Placebo/GH (*n* = 12)				GH/Placebo (*n* = 13)				
	After 1 year placebo		After 1 year GH		After 1 year GH		After 1 year placebo		
Total IQ	61	(55 to 69)	62	(58 to 69)	69	(60 to 73)	70	(57 to 80)	0.832
Verbal IQ	62	(55 to 66)	65	(57 to 70)	66	(57 to 75)	67	(55 to 76)	0.486
- Vocabulary	−2.3	(−2.7 to −2.0)	−2.2	(−2.9 to −2.0)	−2.3	(−3.0 to −1.3)	−2.0	(−2.7 to −1.5)	0.650
- Similarities	−1.8	(−2.2 to −1.7)	−1.7	(−2.3 to −1.1)	−1.3	(−2.3 to −1.0)	−1.3	(−2.3 to −1.0)	0.943
- Arithmetic	−2.0	(−2.2 to −2.0)	−2.0	(−2.0 to −1.7)	−2.0	(−2.0 to −1.7)	−2.0	(−2.0 to −1.8)	0.320
- Digit Span	−2.2	(−2.8 to −1.7)	−2.3	(−2.9 to −1.8)	−2.0	(−2.3 to −1.8)	−2.0	(−2.3 to −1.8)	0.793
- Information	−1.7	(−2.6 to −1.7)	−1.8	(−2.0 to −1.4)	−1.7	(−2.0 to −1.1)	−1.8	(−2.3 to −1.3)	0.154
- Comprehension	−2.3	(−2.7 to −2.3)	−2.2^a^	(−2.6 to −2.0)	−2.0	(−2.6 to −1.4)	−2.0	(−2.6 to −1.4)	0.123
Performance IQ	59	(53 to 72)	57	(52 to 70)	67	(57 to 75)	69	(55 to 78)	0.322
- Picture Completion	−2.2	(−2.9 to −1.7)	−2.5	(−2.9 to −1.7)	−1.7	(−2.8 to −0.8)	−1.3	(−2.3 to −0.4)	0.130
- Coding	−3.0	(−3.0 to −2.2)	−2.7	(−3.0 to −2.3)	−2.5	(−3.0 to −1.5)	−2.3	(−3.0 to −1.5)	0.903
- Block design	−2.0	(−2.2 to −1.4)	−1.5^a^	(−2.0 to −1.3)	−1.7	(−1.8 to −1.2)	−1.7	(−2.0 to −1.0)	0.075
- Matrix Reasoning	−1.7	(−2.1 to −1.3)	−2.3^a^	(−2.3 to −1.7)	−2.2	(−2.3 to −1.2)	−2.2	(−2.3 to −1.3)	0.376
- Picture Arrangement	−2.3	(−2.6 to −1.2)	−2.0	(−2.3 to −1.4)	−1.7	(−1.7 to −1.0)	−1.3	(−2.0 to −1.0)	0.611
FM%	45.3	(38.2 to 48.3)	41.7	(30.6 to 50.6)	39.3^a^	(33.2 to 49.8)	44.1	(38.4 to 52.3)	**0.002**
Lean body mass (kg)	32.3	(30.6 to 45.1)	34.6^a^	(31.6 to 44.0)	35.1	(32.6 to 41.3)	36.7	(31.5 to 39.2)	**0.008**
IGF-I SDS	−0.4	(−0.9 to −0.3)	2.1^a^	(0.0 to 2.4)	1.8^a^	(1.5 to 2.4)	−0.7	(−1.7 to 0.3)	**<0.001**

Data expressed in SDS; median with IQR. *P*-value of mean difference between placebo and GH administration, tested by Wilcoxon tests

^a^within Placebo/GH or GH/placebo group; significantly different compared to placebo

### Associations during placebo

After 1 year of placebo, young adults scored lowest on subtest Coding (−2.7 SDS) and highest on the subtests Similarities, Information and Picture Arrangement (median score −1.7 SDS). After 1 year of placebo, there was no difference in TIQ, VIQ or PIQ between boys and girls (*p* > 0.166) or between patients with a deletion and mUPD + ICD (*p* > 0.138). The IGF-I SDS during placebo was not associated with TIQ, VIQ or PIQ (*p* > 0.602).

### Placebo versus GH administration

Table 2 and Fig. 1 show the effects of 1 year of placebo versus 1 year of GH administration on cognition. Compared with GH treatment, placebo did not deteriorate TIQ, VIQ or PIQ (*p* > 0.322). The difference between placebo and GH administration was strongest in the subtest Block Design, as patients scored 0.3 SDS worse during placebo (median score placebo −2.0 SDS, GH administration −1.66 SDS, *p* = 0.075), but the difference did not reach significance. The young adults had similar scores on the other 10 different subtests during placebo and GH administration (all *p* > 0.123).

The effect of placebo versus GH administration on TIQ, VIQ or PIQ was neither different between boys and girls (*p* > 0.418), nor between young adults with a deletion versus mUPD + ICD (*p* > 0.138). Young adults with a lower TIQ had more loss in TIQ points during placebo versus GH (ρ = −0.407, *p* = 0.043), indicating that if the TIQ is 1 point lower, the difference in TIQ points during placebo versus GH increases with 0.407 points. In particular those with a lower VIQ had more decrease in VIQ points during placebo (ρ = −0.467, *p* = 0.021). The loss in TIQ or VIQ points was not associated with IGF-I SDS during GH or placebo, or age of start GH treatment. In order to further investigate this association, the 8 patients in lowest TIQ tertile were compared with those in the highest tertile. During both GH treatment and placebo, TIQ, VIQ and PIQ were significantly lower in the lowest tertile compared to the highest tertile (all *p* = 0.036). There was no difference in age, BMI, FM%, LBM, IGF-I during placebo or GH, age at start GH treatment, duration GH treatment and GH peak during stimulation test.

Limited cognitive functioning is commonly defined as an IQ score below 70 points [20]. Seven (28%) young adults had a VIQ and 9 (36%) a PIQ higher than 70 points, during both placebo and GH treatment.

### Associations with GH peak during stimulation test

After the 2-year study, twenty-three young adults underwent an arginine-GHRH test. Only 3 (13%) had a GH peak below the BMI-dependent cut-off [21]. There was no significant influence of the GH peak on the effects of placebo versus GH administration on TIQ, VIQ or PIQ (*p* > 0.604).

## Discussion

This is the first 2-year, randomized, double-blind, placebo-controlled GH study in young adults with PWS who were treated with GH during childhood until AH, investigating the effects of cessation of GH (placebo) versus GH administration on cognitive functioning. Our data show that, compared to GH administration, 1 year of placebo did not deteriorate TIQ, VIQ or PIQ in the total group of young adults with PWS. However, patients with a lower cognitive functioning had more loss in IQ points during placebo versus GH treatment.

In this study, we investigated whether the GH-induced improvement in cognitive functioning during childhood would be lost during 1 year of placebo. Our results are reassuring, as there was no significant deterioration in TIQ, VIQ or PIQ after 1 year of placebo in young adults with PWS. If IQ had deteriorated after cessation of GH treatment, this would have suggested that sustained activation with GH was required to retain the improved cognitive functioning achieved during childhood. We found, however, that IQ remained similar after 1 year of placebo, which might indicate that GH treatment during childhood has long-lasting effects. To our knowledge, there are no studies investigating the effects of cessation of GH treatment on cognition after AH attainment.

The scores of the subtest Block Design, however, tended to deteriorate after 1 year of placebo compared to GH treatment, but the decrease of 0.3 SDS was not significant. An RCT in children with PWS showed that 2 years of GH treatment did not significantly improve Block Design scores compared to baseline. Only after 4 years of GH treatment, Block Design scores had increased approximately 0.3 SDS, which was significantly higher than at baseline [6]. Thus, our finding that TIQ did not change during 1 year of placebo compared to GH treatment, while Block Design scores tended to deteriorate, does not exclude that stop of GH treatment for many years could result in a deterioration of cognitive functioning on the long term. It might be that 1 year of placebo is too short to show a significant decrease in cognitive functioning.

There are no other studies on cognitive functioning in young adults with PWS who received long-term GH during childhood. Only one study investigated the effects of GH treatment versus placebo on cognitive functioning in adults with PWS, but these PWS adults were older and GH-untreated at inclusion. They demonstrated that the subtest Block Design and Coding improved during GH treatment and benefits were more pronounced in patients who were GH-treated for the longest time [22]. In untreated adults with PWS, lower IGF-I levels were correlated with poorer intellectual skills [12], which is in line with the beneficial effects of GH treatment, which increases IGF-I. We, however, neither found a correlation between IGF-I SDS during placebo and IQ, nor between IGF-I SDS during GH or placebo and the loss in IQ points. It might be that the beneficial effects of the long-term GH treatment which our patients received during childhood are not nullified during the short time of placebo of 1 year.

In contrast to our current findings of unaltered cognitive functioning after 1 year of placebo versus GH administration, we found an impressive deterioration of the body composition within this period [19]. This might suggest that the beneficial effects on cognition of GH treatment during childhood last into adulthood. The exact mechanism how GH exerts its beneficial effects on cognitive functioning is unknown. It has been suggested that GH may directly affect its GH receptors which are widespread throughout the brain, or through release of IGF-I [23]. The neurotrophic effects of GH continue during adulthood, and it has been proposed that the age-related decline in GH secretion is involved in the decreased neurogenesis in healthy elderly [23]. Amongst them, those with higher IGF-I levels have better cognitive functioning and lower rates of cognitive decline [24, 25]. Besides, an RCT showed that GHRH administration in healthy elderly and in adults with a mild cognitive impairment had favorable effects on cognition [26]. Like in other syndromes, ageing in PWS might occur prematurely, and lower GH levels might be involved in this, but data are very limited [27, 28]. In other syndromes, like Down Syndrome, age-related diseases as dementia with loss of function in multiple cognitive domains are more prevalent and occur earlier [29]. A deterioration in cognitive functioning over time is also seen in patients with Alzheimer’s disease, and GH administration reduced learning and memory deficits in animals with this disease [23]. In adults with GH deficiency, GH administration improved long-term and working memory functions [30, 31]. Altogether, these results support the hypothesis that the GH-IGF-I axis is involved in cognitive functioning [32]. How GH treatment achieves its beneficial effects needs to be elucidated, but it suggests that GH might have neuroprotective effects. Thus, continuation of GH treatment might also benefit adults with PWS, while it cannot be excluded that cessation of GH impairs cognitive functioning on the long-term.

Although there was no deterioration in TIQ, VIQ or PIQ during 1 year of placebo, we found that young adults with a lower cognitive functioning lost more IQ points during placebo versus GH treatment, indicating more benefit from GH treatment. This is in line with our previous finding that GH treatment was more beneficial for children with PWS with lower cognitive functioning [6]. In elderly and in patients with GH deficiency or Alzheimer, there was a positive correlation between IGF-I levels and cognitive functioning, while this was not found in a healthy adult group [33]. The authors postulate that IGF-I levels are not involved in cognition in case of relatively good cognitive performance. This is in line with our findings and suggests that patients with poor cognitive skills are more vulnerable for loss of function. The mechanism is, however, not clear.

Young adults with PWS in our study showed a moderate cognitive impairment, with median IQ scores being about 5 to 10 points higher than documented by other studies, who reported IQ scores between 50 and 60 or around 52 points in adults with PWS [22, 34]. These patients had not received GH treatment during childhood [34]. An explanation for the higher IQ scores in our groups might be that all our participants were treated with GH treatment for many years during their childhood, which is a crucial period for maturation of the brain. GH treatment during childhood prevents deterioration of cognitive skills and improves cognition on the longer term [6]. The patients in this study started GH treatment relatively late at an age of 8.8 years, whereas the children with PWS who are nowadays born start at an earlier age. It can be assumed that GH effects on cognition will be even larger in the future.

Besides the higher IQ scores than reported in the literature, we also found a very wide variation in IQ scores. One patient had a PIQ of 100 during GH and subsequently 92 during placebo, which is exceptionally high for an individual with PWS. Furthermore, the 3^rd^ quartile of TIQ, PIQ and VIQ was higher than the cut-off for intellectual disability [20], meaning that more than 25% of the young adults had an IQ above 70 points. On the other hand, there was a patient who was not able to participate in this part of the study, as she had poor cognitive skills in addition to her hearing impairment. Previously reported percentages of poor cognitive skills in individuals with PWS were around 14% [34, 35], while this was present in only 1 of 27 patients in our group.

Cognition after stop of GH treatment and the effects of placebo versus GH administration on cognition were not different between patients with a deletion and those with an mUPD or ICD. It has been described that patients with an mUPD have better verbal skills than those with a deletion [6, 35]. These patients were, however, not treated with GH. During 4 years of GH treatment, children with an mUPD showed a larger improvement than children with a deletion, resulting in a similar Block Design score as those with a deletion after 4 years of GH treatment [6]. We now found no difference in cognition between those with a deletion versus mUPD + ICD, which suggests that long-term GH treatment during childhood improved cognitive functioning, particularly of those with mUPD + ICD.

The ethical dilemma of 1 year placebo injections in mentally disabled young adults was extensively discussed with patients’ caregivers. The high clinical relevance ensured them to vote for the strongest design, being a 2-year cross-over study. It might be that 1 year placebo was too short to demonstrate alteration in cognitive functioning and that no GH treatment will lead to a deterioration on the longer term. It was, however, considered unethical to extend the placebo period beyond 1 year given the convincing positive effects of GH on body composition. It might be that the effect of GH versus placebo on cognitive skills would have reached significance if we could have studied a larger group. This was, however, not feasible as PWS is a rare disorder.

## Conclusion

In conclusion, this cross-over trial in young adults with PWS who were treated for many years with GH during childhood shows that compared to GH treatment, 1 year of placebo did not deteriorate cognitive functioning. However, patients with a lower cognitive functioning had more loss in IQ points during placebo versus GH treatment. The reassuring finding that 1 year of placebo does not deteriorate cognitive functioning does, however, not exclude a gradual deterioration of cognitive functioning on the long term.
